# Marine Antimalarials

**DOI:** 10.3390/md7020130

**Published:** 2009-04-23

**Authors:** Ernesto Fattorusso, Orazio Taglialatela-Scafati

**Affiliations:** Dipartimento di Chimica delle Sostanze Naturali, Università di Napoli “Federico II”, Via D. Montesano, 49, I-80131, Napoli, Italy. E-Mail: fattoru@unina.it

**Keywords:** Malaria, Marine metabolites, Isonitrile, Alkaloids, Endoperoxides

## Abstract

Malaria is an infectious disease causing at least 1 million deaths per year, and, unfortunately, the chemical entities available to treat malaria are still too limited. In this review we highlight the contribution of marine chemistry in the field of antimalarial research by reporting the most important results obtained until the beginning of 2009, with particular emphasis on recent discoveries. About 60 secondary metabolites produced by marine organisms have been grouped into three structural types and discussed in terms of their reported antimalarial activities. The major groups of metabolites include isonitrile derivatives, alkaloids and endoperoxide derivatives. The following discussion evidences that antimalarial marine molecules can efficiently integrate the panel of lead compounds isolated from terrestrial sources with new chemical backbones and, sometimes, with unique functional groups.

## 1. Introduction

Malaria is an infectious disease caused by several protozoans belonging to the genus *Plasmodium* (*P. falciparum*, *P. ovale*, *P. vivax*, *P. malariae*), with *P. falciparum (Pf)* being the parasite responsible for most severe diseases and most fatal cases. The protozoan comes in contact with humans through the vector contribution of female mosquitoes of the genus *Anopheles*, then it invades red blood cells causing anaemia; the ensuing rupture of infected erythrocytes is associated with the release into the blood stream of cell debris responsible for the characteristic fever spike patterns. In lethal cases, a specific protein produced by the protozoan is embedded into the cell membrane of the infected erythrocyte and, as a consequence of this modification, the erythrocyte sticks to the walls of capillaries causing obstruction of vessels. When this mechanism operates at the level of brain vessels, the loss of consciousness is the first symptom, but, if this form of cerebral malaria is not treated immediately, it is soon followed by death.

The improvement of hygienic conditions, the massive use of insecticides, and the discovery of different drugs played a great role in the nearly complete extinction of infections registered in developed countries. Unfortunately, in the tropical countries of Africa, Asia and America, malaria is still a common cause of death (each year, 300–500 million people become ill with malaria and 1–3 million die) and tragically most of the victims are children under the age of five: every 30 seconds a child dies of malaria [1]. A troublesome increase in the number of fatal cases has been registered in recent years and this is principally due to the spread of multi-drug resistant strains of *Plasmodium*, that make ineffective the limited armamentarium of drugs now available. Since malaria is a disease of worldwide implications and almost half of the world’s population is currently at risk for malaria infection, combating malaria is one of the highest priority programs of WHO [2].

Unfortunately, in spite of several years of efforts and a number of announcements, a vaccine against malaria has not yet been found and the number of different strains causing the pathology and their tendency to genetic mutations promise to create difficulties to this strategy also in the future. Moderate optimism has recently been arisen by RTS, S vaccine, heading phase III trials in 2009, but its potential protective efficacy has been estimated not higher than 30%–40% and only when the vaccine is used in combination with an effective adjuvant therapy [3].

On the other hand, the impressive progresses in the field of genetic research have allowed the sequencing of the entire genome of both the malarial parasite and its insect vector; this important result disclosed a host of pharmaceutical targets to prevent and/or treat the disease [4]. Therefore, the pharmacological therapy has been in the past and currently remains the best answer that researchers have proposed against malaria. Unfortunately, the number of drugs active against malaria continues to be very small and the selection of resistant strains is further narrowing the number of effective molecules. In the last 30 years about 1,700 new drugs have been registered and, among them, only 15 were destined to tropical diseases and only 4–5 against malaria. This picture dramatically illustrates the unquestionable evidence that efforts of pharmaceutical companies are obviously not proportional to the number of deaths for a certain pathology but mostly to the potential market of the developed drugs.

**Figure 1:**
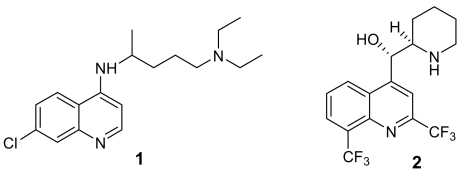
Chloroquine (**1**) and mefloquine (**2**)

The available chemical entities to treat malaria cases are sadly based on very old molecules, like the chloroquine analogues (Figure 1). Although the combination of these molecules with artemisinin and other endoperoxide analogues is giving some good results, the therapeutic choices are still too limited. There is therefore an urgent need for new and innovative drugs. According to Jefford, a drug directed against plasmodium should be active orally for 1–3 days, should give a fast eradication of parasite and disappearance of symptoms, should be safe for children and pregnant women, and, more importantly, should be as cheap as aspirin (<1€ per treatment) [5].

To reach this challenging aim, the identification and selection of new lead compounds constitutes a crucial point. In this regard, living organisms are a recognized source of potentially bioactive molecules which are, commonly, more effective than those obtained through combinatorial synthetic chemistry. Indeed, synthetic libraries are straightforward to assemble, this representing an advantage but also a drawback: the use of a relatively limited number of synthetic reactions as well as of structurally diverse building blocks to library construction implies that combinatorial libraries often lack the structural diversity required for fully exploring the biological space. On the other hand, having been enzymatically engineered and biologically validated, the “quality” of both terrestrial and marine natural products is higher and they represent the ideal tool to harvest the fruits encrypted in a genomic text.

Thus, not surprisingly, the most significant advancements in malaria therapy have been made with the introduction of natural leads. The treatment of malaria infections holds a venerable place both in the history of medicinal chemistry and of natural product chemistry. Indeed, malaria was the first disease to be treated with an active principle isolated from a natural source, quinine, isolated from the Cinchona bark in 1820, and until the 20^th^ century many of the active medicines were developed based on the structure of quinine. A more recent breakthrough in the fight against malaria came with the discovery of artemisinin, an endoperoxide sesquiterpene from *Artemisia annua* [6], an herbal remedy used in Chinese folk medicine. It is now expected that the next significant advancement in the field of antimalarial drugs will not be reached through the discovery of a single potent compound, but through the introduction of an innovative drug to be used in a combined therapy, preferably composed by molecules acting at different stages of the malaria parasite life cycle.

Hopefully, the new breakthrough in the malaria treatment will come with the development of a marine lead compound. The incredible potential of even a single marine organism (mostly invertebrates, as sponges, tunicates, soft corals) to produce a large array of secondary metabolites can be interpreted by considering the common features of the secondary metabolism in all the living organisms as well as some peculiar features of the marine environment. In addition, the contribution of the symbiotic population to the metabolic work of a marine invertebrate is an important point to be taken into account. Indeed, marine invertebrates harbor in their tissues a series of microorganisms such as bacteria, cyanobacteria and fungi and, in some cases, associated micro-organisms may constitute up to 40% of the biomass [7,8], this bacterial concentration exceeding that of the surrounding sea water by two or three orders of magnitude. Although the real contribution of the microorganisms to the secondary metabolism of marine invertebrates has not yet been fully evaluated, essentially because of the difficulties encountered in culturing sponge-associated bacteria, it is generally accepted that these harbored microorganisms play a significant role in the biosynthesis of the natural products isolated from the invertebrate. Furthermore, the recent impressive advances in molecular genetics, currently allowing the identification of biosynthetic genes in the producing organisms and their cloning in bacteria suitable for large-scale fermentation [9] could represent a solution for the problem of compound supply. If these techniques will be fully developed and utilized, the last obstacle to consider marine organisms as a potentially sustainable drug source would be overcome.

This review aims at highlighting the contribution of marine chemistry in the field of antimalarial research, by reporting the most important results obtained until the beginning of 2009, with particular emphasis on recent discoveries. We have decided to include in this review all those compounds possessing a moderate to high antimalarial activity, thus excluding very weak antimalarials or molecules for which the toxicity toward *Plasmodium* strains is not specific and/or is clearly due to a general cytotoxicity. An interesting review has been recently published focusing on the synthesis of marine natural products with antimalarial activity [10]; consequently, we will not discuss in detail synthetic efforts aimed at preparing marine antimalarial leads.

Following a scheme that we have introduced in a recent book chapter [11], marine antimalarials have been divided throughout this review, according to their chemical structures, into three different classes: i) isonitrile-containing derivatives; ii) alkaloids; iii) endoperoxides. A paragraph including miscellaneous compounds not belonging to the above classes is presented at the end of the review.

## 2. Isonitrile-containing derivatives and their analogues

**Figure 2:**
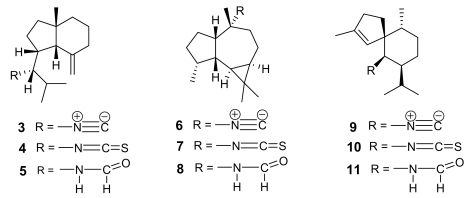
Isonitrile-, isothiocyanate- and formamide-containing sesquiterpenoids from the sponge *Axinella cannabina.*

The parent compound of the small class of isonitrile-containing marine secondary metabolites is axisonitrile-1 (**3**, Figure 2), isolated in 1973 from the marine sponge *Axinella cannabina*, where it co-occurred with the strictly related axisothiocyanate-1 (**4**) [12]. Axisonitrile-1 was soon followed by other isonitrile-, isothiocyanate-, and formamide-containing sesquiterpenoids from the same source, namely axamide-1 (**5**), axisonitrile-2, (**6**) [13], axisothiocyanate-2 (**7**), axamide-2 (**8**) [14], axisonitrile-3 (**9**), axisothiocyanate-3 (**10**), and axamide-3 (**11**) [15] (Figure 2).

The chemical structure of these derivatives has been confirmed by elegant syntheses, including the very recent total synthesis of axamide-1 and axisonitrile-1 via 6-*exo*-dig radical cyclization [16]. In 1992, axisonitrile-3 (**9**) was re-isolated from the sponge *Acanthella klethra* Pulitzer-Finali and found to possess a potent antimalarial activity both on chloroquine-sensitive (D6, 142 ng/mL) and chloroquine-resistant (W2, 17 ng/mL) *P. falciparum* strains [17], and to be practically devoid of cytotoxicity toward KB cells. The closely related axisothiocyanate-3 (**10**) was inactive, suggesting that the antiplasmodial activity should not (or, at least, not only) be ascribed to structural features of the carbon backbone but should be strictly dependent on the presence of the isonitrile functional group.

**Figure 3:**
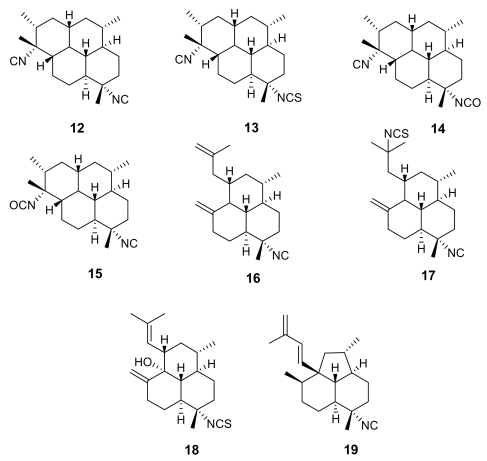
Representative isonitrile- and isothiocyanate-containing diterpenoids isolated from the marine sponge *Cymbastela hooperi.*

These remarkable findings stimulated an in-depth research activity aimed at isolating and testing isonitrile secondary metabolites from different marine sources, with particular regards to marine sponges of the families Axinellidae and Halicondridae appearing to selectively elaborate these kinds of metabolites. The chemical analysis of the sponge *Cymbastela hooperi* (Axinellidae) afforded a series of diterpenes based on amphilectane, isocycloamphilectane, and neoamphilectane skeletons and bearing isonitrile, isothiocyanate, and the rare isocyanate functionalities (Figure 3) [18]. These molecules displayed a significant (low nM range) and selective (cytotoxicity in the μM range) *in vitro* antimalarial activity and the co-occurrence of several strictly related analogues suggested some structure-activity relationships.

Comparison among the activities exhibited by the closely related isocycloamphilectanes **12** (IC_50_ ~ 4 ng/mL), **13** (IC_50_ ~ 40 ng/mL), and **14** (IC_50_ ~ 60 ng/mL) illustrated the relative potency of isonitrile, isothiocyanate and isocyanate groups, supporting the previous observation that the bioactivity is particularly associated to the presence of the isonitrile group. However, the location of functional groups also plays a role, as suggested by the comparison between the activities of compounds **14** and **15** (IC_50_ ~ 3 ng/mL), where the positions of isocyanate and isonitrile groups are interchanged.

The importance of the isonitrile group is highlighted by the relatively low activity of the amphilectane isothiocyanate **18** (IC_50_ ~ 800 ng/mL), and by the similarly low activity of amphilectane formamides, very recently isolated from the same source [19]. On the other hand, the activity of the neoamphilectane derivative **19** (IC_50_ = D6, 90 ng/mL; W2, 30 ng/mL) is considerably higher than that of compound **16**, indicating that the carbon skeleton can modulate the antiplasmodial activity of isonitrile derivatives.

**Figure 4:**
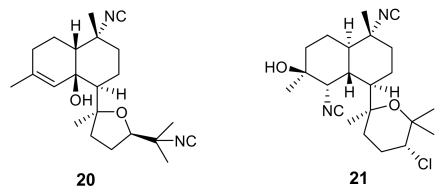
Representative kalihinane diterpenoids isolated from the marine sponge *Acanthella* sp.

Further isonitrile-containing antimalarial derivatives have been isolated from the Japanese sponge *Acanthella* sp. (e.g. **20–21**, Figure 4) [20]. These molecules belong to the class of the kalihinane diterpenoids, which comprises also antifungal, anthelmintic and antifouling compounds. Isonitrile kalihinanes showed a potent antiplasmodial activity in the very low nanogram range [e.g. kalihinol A (**21**) IC_50_ = 0.4 ng/mL]. A total synthesis of a kalihinol A analogue has been accomplished [21].

A hybrid modelling technique combining 3D-QSAR with quasi-atomistic receptor modelling methodologies has been used to generate a pharmacophore hypothesis for isonitrile derivatives consistent with the experimental bioactivities, indicating a likely mechanism of action for this class of marine antimalarials [22]. Active isonitriles were demonstrated to interact with free heme by forming a coordination complex with the iron center; the “pharmacophore” must possess an overall lipophilic rigid molecular core comprising at least a tricyclic framework carrying an isonitrile group and establishing further hydrophobic interactions above the ring plane. Interaction of marine isonitriles derivatives with heme could inhibit the transformation of heme into *β*-hematin and then hemozoin, a polymer produced by *Plasmodium* in order to neutralize the toxic (detergent-like) free heme produced in the food vacuole. In addition, isonitriles were shown to prevent both the peroxidative and glutathione-mediated destruction of heme under conditions that mimic the environment within the malaria parasite. Thus, isonitriles exert their antiplasmodial activity by preventing heme detoxification [22].

## 3. Alkaloids

**Figure 5:**
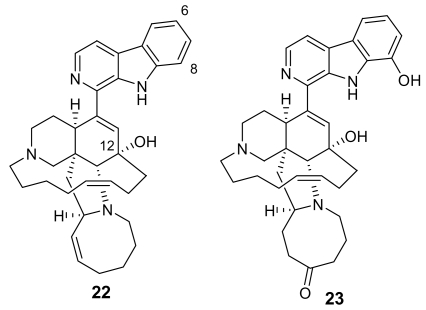
Chemical structure of manzamine A (**22**) and manzamine F (**23**)

Manzamines are undoubtedly the most important and potent antimalarial alkaloids isolated from marine sources. They are very complex polycyclic (7–8 rings or more) alkaloids first reported by Higa and coworkers in 1986 from an Okinawan sponge belonging to the genus *Haliclona* [23,24]. These molecules are characterized by an intricate pentacyclic heterocyclic system attached to a β-carboline moiety. Since the first report of manzamine A (**22**, Figure 5), at least 60 additional manzamine-type alkaloids have been reported from taxonomically unrelated sponges belonging to different genera (e.g. *Xestospongia*, *Ircinia*, and *Amphimedon*) and different orders. These findings strengthen the hypothesis that manzamines are not true sponge metabolites but, more likely, they have a symbiotic origin. Accordingly, microbial community analyses for one of the most common manzamine producing sponges resulted in the identification of *Micronosphora* sp. as the bacteria producing manzamines [25]. A recent paper hypothesized a common biosynthetic route for cyclostellettamines, halicyclamines and manzamines, thus highlighting the existence of “nature diversity-oriented syntheses” [26].

Fourteen years after its first isolation, Hamann *et al*. disclosed the antimalarial potential of manzamine A [27]. This molecule, and its 8-hydoxy derivative, were found to potently inhibit the growth of *Plasmodium falciparum* both *in vitro* (IC_50_ ~ 5.0 ng/mL) and *in vivo* (a parasitemia suppression of the same order of magnitude of that of artemisinin). Unfortunately, the therapeutic index of these molecules is somewhat narrow (gastrointestinal distress) and further studies are needed to improve this value.

Interestingly, a closely related derivative of manzamine A, manzamine F (**23**, Figure 5), is completely devoid of activity (IC_50_ > 1,000 ng/mL), thus evidencing the key role of the eight membered ring, where the differences between the inactive manzamine F and the active manzamine A are confined. The reduction of the double bond and/or the insertion of a ketone group on the adjacent carbon is evidently deleterious for the antimalarial activity. Similarly, the attachment of the hydroxyl group at position 6, in place of position 8, has a deleterious impact on the antimalarial activity, as indicated by the lower potency of manzamine Y (6-hydroxy-manzamine A), exhibiting IC_50_ ~ 600 ng/mL [28].

**Figure 6:**
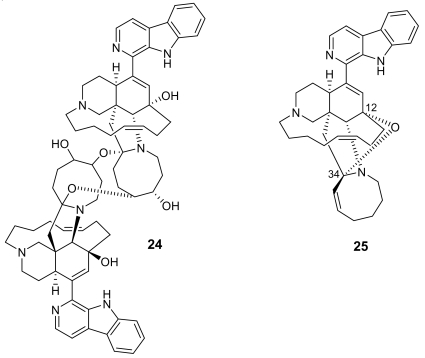
Chemical structures of *neo*-kauluamine (**24**) and 12,34-oxamanzamine A (**25**).

Additional information on structure-activity relationships came with the isolation of *neo*-kauluamine (**24**, Figure 6) a very complex molecule constituted by two manzamine units dimerized through ether linkages between the eight-membered rings [29]. Although this molecule, like manzamine F (**23**), lacks the double bond in the eight-membered ring, it demonstrated the same antimalarial activity as manzamine A. The lack of antimalarial activity for 12,34-oxamanzamine A (**25**, Figure 6) (IC_50_ = 5 μg/mL) [30] indicates that the C-12 hydroxyl, the C-34 methine or the conformation of the eight-membered ring are of key importance for the antimalarial activity. The crucial role of the hydroxy group at position 12 has also been highlighted by a recent study reporting that acetylation at position 12 significantly reduced the antimalarial activity [31].

Manzamines have also been reported to be antiinflammatory, antifungal, antibacterial and antitubercolosis agents, and to exhibit activity against AIDS opportunistic pathogens (e.g. *Toxoplasma gondii*) [29, 32, 33]. More recently, inhibitory activity against glycogen synthase kinase-3 (GSK-3) has been discovered for manzamine A and derivatives [34]. The inhibited kinase is involved in pathological hyperphosphorylation of protein tau and, therefore, manzamine A constitutes a promising scaffold to design potential therapeutic agents for Alzheimer’s disease. The highly complex structure of manzamines have made them an attractive total synthesis target, and three different total syntheses have been published [35–37].

**Figure 7:**
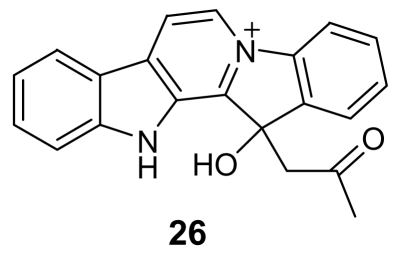
Chemical structure of homofascaplysin A (**26**)

The *β*-carboline group is also present in the structure of homofascaplysin A (**26**, Figure 7), isolated from the sponge *Hyrtios erecta* [38]. This alkaloid showed activity against chloroquine-resistant *P. falciparum* strains with an IC_50_ of about 20 ng/mL, but its toxicity toward rat skeletal muscle myoblast cells was estimated to be less than 1 μg/mL.

**Figure 8:**
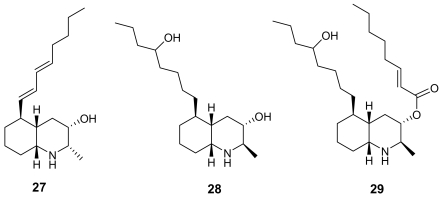
Chemical structures of lepadins B (**27**), D (**28**) and E (**29**).

Lepadins are decahydroquinoline derivatives bearing a linear eight-carbon chain obtained from two marine invertebrates of Australian origin, *Clavelina lepadiformis* [39] and *Didemnum* sp. [40]. Lepadin E (**29**, Figure 8) exhibited a significant antimalarial activity (IC_50_ = 400 ng/mL) while its close analogues lepadin B (**27**) and D (**28**) (Figure 8) are almost completely inactive. This marked difference of activity highlights the importance of the 2*E*-octenoic acid ester functionality in place of the secondary alcohol. Authors have proposed that this conformationally mobile side-chain could serve to stabilize non-bonding interactions with heme, or with any other “receptor” molecule. On the other hand, lepadin B has been found to block neuronal nicotinic acetylcholine receptors [41]. A number of total syntheses of lepadins have been accomplished [42].

Phloeodictynes are a class of marine alkaloids whose chemical structure appears to be related to that of lepadins in having a bicyclic nitrogen-containing skeleton bearing a long alkyl chain. Phloeodictynes, isolated from sponges belonging to the genus *Oceanapia* [43, 44], are 1,2,3,4-tetra-hydropyrrolo-[1,2-*a*]-pyrimidinium derivatives bearing at C-6, in addition to an OH group, a variable-length alkyl chain and at N-1 a four/five methylene chain ending in a guanidine group.

**Figure 9:**
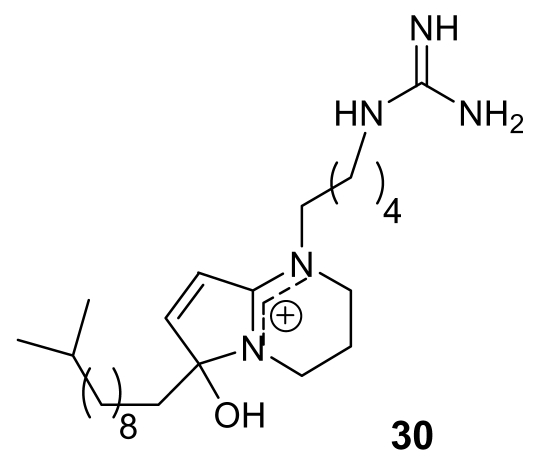
Chemical structure of the active compound phloeodictyn 5,7i (**30**).

Phloeodictyn 5,7i (30, Figure 9) exhibited a good activity (IC50 = 300 ng/mL) against the chloroquine-resistant FGB1 strain of the malaria parasite *Plasmodium falciparum*, with a good therapeutic index [44]. Total synthesis for these molecules has been reported [45].

**Figure 10:**
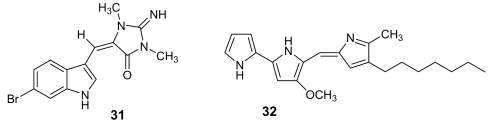
Chemical structures of 6-bromoaplysinopsin (**31**) and of heptylprodigiosin (**32**).

6-Bromoaplysinopsin (31, Figure 10) is a simple indole derivative first isolated in 1985 [46] and later re-obtained from the sponge *Smenospongia aurea* [47], active against the D6 clone of *P. falciparum* with IC_50_ = 340 ng/mL and a low selectivity index. Heptyl prodigiosin (**32**, Figure 10) is a pigment, purified from a culture of α-proteobacteria isolated from a marine tunicate, which showed an antimalarial activity similar to that of quinine against the chloroquine–sensitive strain *P. falciparum* 3D7 with an *in vitro* activity that was about 20 times the *in vitro* cytotoxic activity against mouse lymphocytes. When this molecule was tested *in vivo*, a single administration of 5 mg/kg significantly extended the survival of *P. berghei* ANKA strain-infected mice but, unfortunately, the same dose caused sclerotic lesions at the site of injection [48].

All the above marine alkaloids are characterized by a relatively poor knowledge of the mechanism of their antimalarial action, which is quite important to develop more potent and/or structurally simplified analogues. On the other hand, a couple of recently published antimalarial marine alkaloids have been found in the frame of investigations addressed against specific parasite targets.

**Figure 11:**
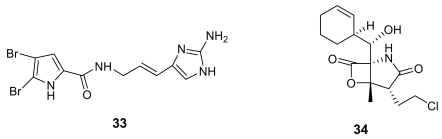
Chemical structures of oroidin (**33**) and salinosporamide A (**34**).

Oroidin (**33**, Figure 11), the parent compound of a well-known class of marine alkaloids [49, 50], has been found to inhibit *Plasmodium falciparum* enoyl-ACP reductase (IC_50_ = 0.30 μg/mL), an enzyme involved in the parasite fatty acid biosynthesis [51]. Further studies on the numerous oroidin analogues could reveal the structural requirements to interact with this target.

Salinosporamide A (**34**, Figure 11) is a γ-lactam alkaloid isolated from a marine bacterium of the new genus *Salinispora* [52]. This molecule has been found to be a potent parasite proteasome inhibitor and to possess a quite significant antimalarial activity *in vitro* (IC_50_ = 11.4 nM) [53]. Salinosporamide was determined to act in the erythrocytic stage and maintained its potent activity in a malaria mouse model with inhibition of the parasite growth in treated mice at extremely low doses (130 μg/kg).

Although some total syntheses of salinosporamide have been reported [54, 55], as for manzamine A, the bacterial origin of salinosporamide should provide opportunities for a renewable supply of the compound without harvesting large quantities of marine invertebrates. Anyway, in this case, also the relatively simple chemical structure of salinosporamide A could play in favour of the further development of this molecule as antimalarial drug.

## 4. Endoperoxides

**Figure 12:**
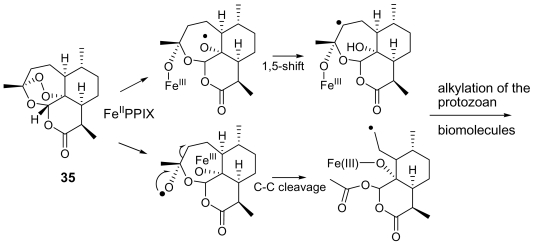
Chemical structure of artemisinin (**35**) and postulated mechanism of its action.

One of the more substantial breakthroughs in malaria chemotherapy has been the discovery and development of endoperoxide-containing drugs. Research in this field began with the discovery that artemisinin (**35,** Figure 12), an endoperoxide cadinane sesquiterpene lactone possessing a 1,2,4-trioxane moiety, isolated from *Artemisia annua* (Compositae) leaves, possessed nanomolar activity also against chloroquine-resistant strains of *Plasmodium* [6, 56]. With its unique juxtaposition of peracetal, acetal and lactone functionalities, artemisinin possesses structural features very appealing to organic chemists. Totally synthetic routes to artemisinin have been developed [57], but their complexity suggests that they will very unlikely supplant the natural extract as a drug supply.

The endoperoxide linkage is an essential feature for antimalarial activity of artemisinin and of its derivatives (e.g. the oil-soluble artemether, the water-soluble artesunate). Indeed, according to the widely accepted mechanism of action, these molecule interact with the iron(II) center of the heme unit released during the digestion of hemoglobin. This yields to the cleavage of the peroxide bridge and to the consequent formation of oxygen-centered radicals which, after an intramolecular rearrangement, convert into free C-centered radicals. These should be toxic to the parasite through alkylation of “sensitive” macromolecular targets (Figure 12). A Ca^2+^-dependent ATPase specific of *P. falciparum* (PfATP6) has been suggested as a potential target for these active species [58].

Intense research activity is currently ongoing in many laboratories around the world in order to obtain from natural sources endoperoxide derivatives which could constitute a valuable alternative to artemisinin. In this context, the next two sections will provide a survey of the contribution in this field coming from marine chemists. For clarity, these molecules have been divided in two categories according to their postulated (and only in few cases unambiguously demonstrated) biogenetic origin: polyketides and terpenoids.

### 4.1. Polyketides

Marine sponges belonging to the family Plakinidae contain a series of simple endoperoxide derivatives that have been identified as polyketide metabolites possessing six- or five-membered 1,2-dioxygenated rings (1,2-dioxane or 1,2-dioxolane, respectively). A further variation is represented, in some cases, by the presence of a 3-methoxy substitution, building a peroxyketal group.

**Figure 13:**
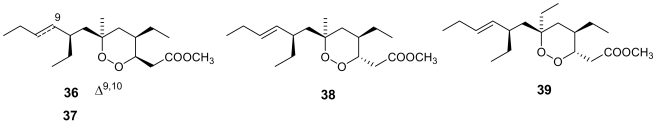
Chemical structures of plakortin (**36**) dihydroplakortin (**37**), 3-epiplakortin (**38**), and plakortide Q (**39).**

Plakortin (**36**, Figure 13) was isolated more than 25 years ago from *Plakortis halichondroides* [59] and recently re-isolated in remarkable amounts from the Caribbean sponge *Plakortis simplex* [60]. In the latter study the absolute configuration of the four stereogenic carbons of plakortin has been determined by means of chemical derivatization and reaction with chiral auxiliaries. The plakortin analogues, dihydroplakortin (**37**), 3-epiplakortin (**38**), plakortide Q (**39**) (Figure 13) have been obtained from the same sponge [60,61].

All these molecules exhibited a good antimalarial activity against D10, chloroquine-sensitive strain, and W2, chloroquine-resistant strain of *P. falciparum*, with a more potent activity on the W2 strain (IC_50_ ~ 180 ng/mL), devoid of cytotoxicity [62]. The chemical structure of these antimalarial leads is remarkably simple and they could constitute a good probe to investigate the mechanisms of action as well as structure-activity relationships. In this regard, a series of semisynthetic derivatives of plakortin (**37**) has been prepared [63] and their activity highlighted the crucial roles both of the “western” alkyl side chain and of the conformational behaviour of the dioxane ring in the light of the interaction with the heme planar target.

Furthermore, strong evidence have been gathered about the plakortin production by a bacterial symbiot of the sponge *Plakortis simplex* [64]. Hopefully, an approach based on identification, cloning and expression of the gene cluster(s) for plakortin and related compounds from the metagenome of the sponge could allow the production of plakortin by easy and low-cost bacterial fermentation, thus fulfilling one of the essential requirements of next generation antimalarial drugs.

**Figure 14:**
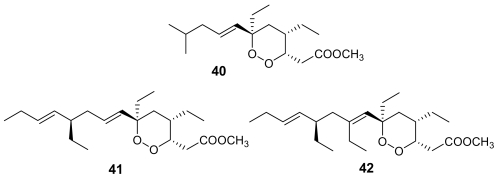
Chemical structures of plakortides L (**40**), O (**41**) and P (**42**).

Interestingly, plakortide L (**40**, Figure 14), isolated from a Jamaican sponge *Plakortis* sp. [65], plakortide O (**41**) and plakortide P (**42**), isolated from *Plakortis halichondrioides* [66], showed a very low *in vitro* antimalarial activity (IC_50_ > 8 μg/mL), in spite of their similarity with the plakortin scaffold. The configurational changes around the dioxane ring and/or the differences in the alkyl side chains are evidently responsible for the marked decrease of activity.

**Figure 15:**
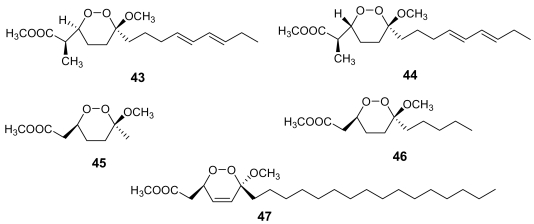
Chemical structures of peroxyplakoric acids A_3_ (**43**) and B_3_ (**44**) methyl esters, of chondrillin (**47**) and of two synthetic analogues (**45** and **46**).

A structurally related class of marine metabolites showing a good antimalarial activity is that of 3-alkoxy-1,2-dioxane (peroxyketals) derivatives. In this class of molecules, the alkoxy substituent at position 3 could partly mimic the non-peroxidic oxygen atom of the 1,2,4-trioxane ring of artemisinin. The methyl esters of peroxyplakoric acids A_3_ (**43**, Figure 15) and B_3_ (**44**, Figure 15) isolated from *Plakortis* sp., showed IC_50_ = 50 ng/mL against *P. falciparum* with a good selective toxicity index (about 200) [67].

Through the syntheses of some analogues of these active compounds, some conclusions about the structural requirements within this class of antimalarials were drawn. For example, compound **45** (Figure 15) proved to be almost completely inactive, whereas compound **46** (Figure 15) retained the *in vitro* activity of peroxyplakoric acid B_3_ methyl ester, indicating the importance of the side chain for the antimalarial activity [68]. Furthermore, it has been demonstrated that the *in vivo* antimalarial potency of these compounds is increased by transforming the ester group into an amide group [69].

Finally, the low antimalarial activity observed for a marine endoperoxide strictly related to peroxyplakoric acid B_3_ methyl ester, namely chondrillin, (**47**, Figure 15) [70] indicates that the presence of a double bond within the 3-methoxy-1,2-dioxane skeleton is detrimental for the activity, although the role of the long saturated alkyl chain should also be taken into account.

### 4.2. Terpenoids

**Figure 16:**
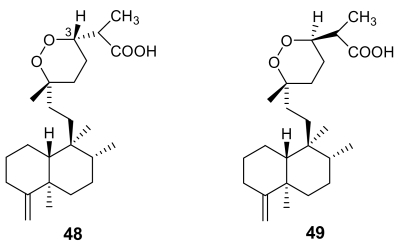
Chemical structures of sigmosceptrellin A (**48**) and of its C-3 epimer sigmosceptrellin B (**49**).

Unfortunately, very few endoperoxide-containing terpenoids isolated from marine sources have been tested for their antimalarial activity. Sigmosceptrellin A, (**48**, Figure 16), is a norsesterterpene derivative active against *P. falciparum* (IC_50_ ~ 450 ng/mL) [71]. Interestingly, the C-3 epimer of **48**, named sigmosceptrellin B, (**49**) proved to possess an activity about four times lower in the same test [72]. This is a good demonstration of the importance of stereochemistry to determine the antimalarial activity in the series of 1,2-dioxane derivatives.

**Figure 17:**
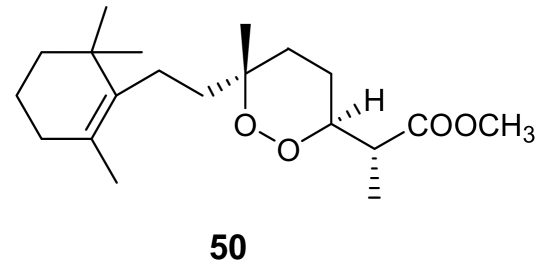
Chemical structure of methyl-3-epinuapapuanoate (**50**).

Methyl-3-epinuapapuanoate, (**50**, Figure 17), a norditerpene derivative isolated from the New Caledonian sponge *Diacarnus levii* [73], showed a moderate *in vitro* activity against chloroquine-resistant strains of *P. falciparum* (IC_50_ = 1.2 μg/mL) [74]. When the molecule was tested against *P. berghei in vivo*, at the concentration of 25 mg/kg, a 56% growth inhibition was observed.

## 5. Miscellaneous Compounds

In this section we have grouped all the marine secondary metabolites endowed with antimalarial activity and not falling in one of the preceding groups, namely they do not contain an isonitrile or an endoperoxide group and they are not alkaloids. These molecules can be further divided into two structural categories, namely quinones and phenols, and peptides.

### 5.1. Quinones and Phenols

**Figure 18:**
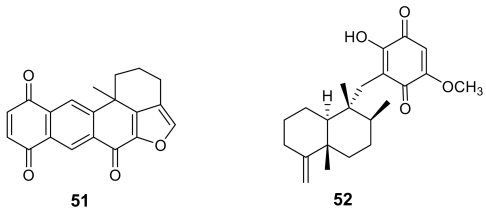
Chemical structures of xestoquinone (**51**) and ilimaquinone (**52**).

In the frame of a search for *Plasmodium* protein kinase inhibitors, the previously known xestoquinone (**51**, Figure 18) [75] proved to be a selective active inhibitor of Pfnek-1, a never-in-mitosis/*Aspergillus* (NIMA)-related protein kinase of *P. falciparum* [76]. However, the *in vitro* antiplasmodial activity of xestoquinone was moderate (IC_50_ ~ 3 μM). A similar activity was exhibited by another marine quinone, ilimaquinone (**52**), isolated from the Australian marine sponge *Dactylospongia elegans* [77].

**Figure 19:**
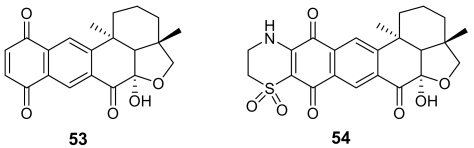
Chemical structure of alisiaquinones A (**53**) and C (**54**).

**Figure 20:**
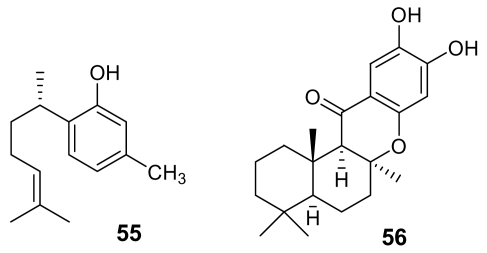
Chemical structures of cucurphenol (**55**) and 15-oxopuupehenol (**56**).

A series of quinone derivatives, named alisiaquinones (e.g. alisiaquinone A, **53**, Figure 19) structurally related to xestoquinone, have been isolated from an unidentified New Caledonian sponge [78]. Not surprisingly they showed an activity against Pfnek-1 and a micromolar antiplasmodial activity similar to those exhibited by xestoquinone. However, quite interestingly, alisiaquinone C (**54**), bearing an additional heterocycle formed by a taurine substituent, exhibited a submicromolar activity (IC_50_ ~ 0.1 μM) on *P. falciparum* and a competitive selectivity index on the different plasmodial strains. This higher activity of alisiaquinone C (**54**) resulted to be not related to a more potent inhibition of Pfnek-1 but to a significant inhibition of the plasmodial enzyme farnesyl transferase.

(*S*)-Curcuphenol (**55**, Figure 20) is a sesquiterpene phenol isolated from different marine sponges belonging to the genus *Didiscus* [79]. This molecule exhibited *in vitro* antimalarial activity with MIC of 3.6 μg/mL against the D6 clone of *P. falciparum* and of 1.8 μg/mL against the W2 clone. Another phenol-containing antimalarial marine metabolite is 15-oxopuupehenol (**56**), isolated from sponges of the genus *Hyrtios*, and representative of a distinctive family of sponge metabolites comprising also the quinol-quinone pair of avarol and avarone. Compound **56** exhibited *in vitro* activity against *P. falciparum* with MIC of 2.0 μg/mL against the D6 clone of *P. falciparum* and of 1.3 μg/mL against the W2 clone [80].

### 5.2. Peptides

**Figure 21:**
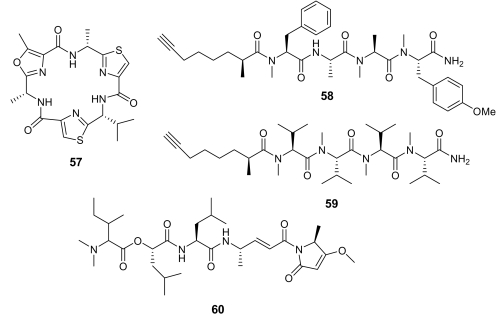
Chemical structures of venturamide A (**57**), dragomabin (**58**), dragonamide B (**59**), and gallinamide A (**60**).

Two modified cyclic hexapeptides, venturamides (e.g. venturamide A, **57**, Figure 21), isolated from the marine cyanobacterium *Oscillatoria* sp., exhibited a moderate activity against *Plasmodium falciparum* (IC_50_ ~ 6–7 μM) [81], with a good selectivity.

A similar antimalarial profile was also exhibited by dragomabin (**58**, Figure 21), a linear alkynoic lipopeptide isolated from a Panamanian strain of the marine cyanobacterium *Lyngbya majuscula* [82]. Interestingly, the related non-aromatic dragonamide B (**59**, Figure 21), isolated from the same source, was completely devoid of activity.

A further cyanobacterial peptide derivative, named gallinamide A (**60**, Figure 21) has been isolated from *Schizothrix* species. This highly functionalized peptide, containing the very unusual 4-(*S*)-amino-2-(E)-pentenoic acid subunit and an *N, N*-dimethyl isoleucine terminus, exhibited a moderate *in vitro* antimalarial activity (IC_50_ = 8.4 μM) [83].

**Figure 22:**
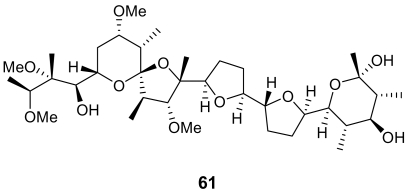
Chemical structure of the polyether **61.**

Finally, a secondary metabolite belonging to a completely different class, a polyether (**61**, Figure 22) obtained from the marine *Streptomyces* sp. H668, exhibited a significant *in vitro* antimalarial activity (IC_50_ ~ 150 ng/mL), with a good selectivity [84]. Some examples of non-marine antimalarial polyether compounds are also present in the literature [85]. These compounds are believed to act as ionophores, and, due to their lipophilic character they are particularly able to interact with the protozoan- infected cell membrane.

## 6. Conclusions

Data reported in the preceding paragraphs clearly demonstrate that the marine environment contains a number of compounds that could serve as lead structures for the development of new classes of antimalarial drugs. The antimalarial marine molecules can efficiently integrate the panel of lead compounds isolated from terrestrial sources (as in the case of endoperoxide derivatives) with new chemical backbones and, sometimes, with unique functional groups (as in the case of isonitrile derivatives). Of course, the drug potential of the about 60 molecules reported in this review is not the same, being related to the potency, to the selectivity, to the toxicity, and to the availability of the compounds. Among them, some isonitrile containing diterpenes, manzamines, salinosporamide, and some polyketide endoperoxides could be indicated as the most promising candidates to future developments. In this regard, the possibility of producing these molecules through bacterial fermentation combined with genetic engineering is a key issue to increase the chances of their full pharmacological evaluation and their possible introduction in therapy.
